# A Comprehensive NMVOC Speciation Database for European Atmospheric Modelling

**DOI:** 10.1038/s41597-026-07538-z

**Published:** 2026-06-01

**Authors:** Kevin Oliveira, Marc Guevara, Jeroen Kuenen, Oriol Jorba, Carlos Pérez García-Pando, Hugo Denier van der Gon

**Affiliations:** 1 Barcelona Supercomputing Center, Plaça Eusebi Güell 1-3, Barcelona, 08034 Spain; 2 Department of Climate, Air and Sustainability, TNO, Princetonlaan 6, Utrecht, 3584 CB The Netherlands; 3 ICREA, Catalan Institution for Research and Advanced Studies, Barcelona, 08010 Spain

## Abstract

Speciated non-methane volatile organic compound (NMVOC) emissions are essential for air quality modelling, as they underpin atmospheric oxidation processes, secondary pollutant formation, and the evaluation of mitigation strategies. Yet, these emissions remains poorly quantified in Europe. Here, we introduce a new database of NMVOC speciation profiles that prioritises European-specific data consistent with present-day technologies, fuels, and regulatory frameworks, and covers all anthropogenic emission sources reported by European countries. The database includes 130 chemical speciation profiles and over 900 individual NMVOC species, systematically compiled and harmonised from the literature. To facilitate integration within official emission inventories, the profiles are mapped to the Selected Nomenclature for Air Pollution (SNAP) and Nomenclature for Reporting (NFR) sector classifications. Each profile provides species-level mass fractions and their aggregation into the 25 Global Emissions Inventory Activity (GEIA) NMVOC groups, enabling straightforward integration with chemical mechanisms used in contemporary air quality models. In addition, a ready-to-use file compatible with the Copernicus Atmosphere Monitoring Service European regional emission inventory (CAMS-REG) is provided.

## Background & Summary

Non-methane volatile organic compounds (NMVOC) comprise a broad and chemically diverse group of species that vary widely in structure, reactivity, and atmospheric oxidation pathways^1^. Emitted from both anthropogenic and biogenic sources, NMVOCs adversely affect human health and play an important role in atmospheric chemistry, as their oxidation drives the formation of ozone (O_3_) and secondary organic aerosols (SOA)^2–6^. Through these processes, NMVOC emissions directly influence compliance with the new European Ambient Air Quality Directive (2024/2881/EU)^7^, which introduces more stringent limit values for O_3_ and particulate matter (PM). The directive further recommends the monitoring of 45 individual NMVOC species; however, information on their spatial distribution and emission sources is at present very limited.

Despite their importance, NMVOC emissions at the individual species level are poorly characterised in Europe. Under the Convention on Long-Range Transboundary Air Pollution (CLRTAP) and the National Emission Ceilings Directive (NECD), EU Member States are required to report only total NMVOC emissions. Source-specific speciation profiles—defined as the mass fractions of individual NMVOC species emitted from a given source, fuel, or technology—are not officially reported, and relatively few efforts have been made to update or harmonise them^8^. As a result, European air quality modelling continues to rely primarily on speciation data developed in the 1990s^9,10^, a limitation that propagates into major emission inventories^11–13^. While emission characteristics in some sectors may have remained stable, many others have undergone substantial changes due to technological advances (e.g., cleaner vehicle fleets under successive EURO standards and cleaner fuels) and regulatory measures (e.g., solvent use and industrial emission directives)^14,15^. The continued use of outdated or non-representative speciation profiles can therefore introduce large uncertainties into emission estimates and air quality simulations^16–19^.

In contrast, other regions have invested significant efforts in refining and updating sector-specific NMVOC speciation profiles. In the United States, the Environmental Protection Agency (EPA) maintains the SPECIATE database, which contains a vast number of local profiles and is continuously updated^20,21^. In Asia, particularly in China, growing concerns over O_3_ pollution have led to extensive field measurement campaigns targeting a wide range of NMVOC emission sources, resulting in the development and regular updating of several regional speciation databases^22–24^.

Detailed compound-level NMVOC emissions are essential for atmospheric modelling. Explicit chemical mechanisms, such as the Master Chemical Mechanism (MCM) and the Generator of Explicit Chemistry and Kinetics of Organics in the Atmosphere (GECKO-A), resolve NMVOC oxidation pathways and therefore require detailed compound-level information^25–27^. Regional air quality models, by contrast, rely on simplified chemical schemes to reduce computational demand. These schemes represent NMVOCs using lumped species that group chemically similar compounds, often starting from individual species or, in some cases, from the 25 NMVOC groups defined by the Global Emissions Inventory Activity (GEIA)^11^. These groups are subsequently mapped onto the species defined within a given chemical mechanism. Chemical mechanisms vary widely among models and often exist in multiple versions or adapted forms^28^. Although efforts have been made to facilitate cross-mechanism mapping for NMVOC emissions^29^, such resources are not always readily available or straightforward to implement, and may omit less abundant but chemically relevant species, thereby introducing additional uncertainty^30^.

To address these gaps, we present a harmonised compilation of NMVOC speciation profiles designed for emission and air quality modelling applications in Europe. The database comprises 130 speciation profiles and more than 900 individual NMVOC species, systematically compiled and standardised to replace inconsistent and outdated information. Wherever possible, profiles were selected to reflect current emission characteristics based on European technologies, fuels, and regulatory conditions. By combining profiles from multiple sources, the dataset provides detailed species-level information across all major anthropogenic emission European sectors. Earlier versions of this database have been applied and evaluated for selected NMVOC species (including benzene, toluene, and xylene) in Spain and at the European scale through comparisons of model results with observations^19,31,32^.

The present release provides three main data products. The first two files contain an archive of 130 chemical profiles describing the species composition of total NMVOC emissions in terms of mass fractions. One file provides the split of total NMVOC emissions by individual chemical species (over 900 NMVOC species), while the second aggregates emissions into the 25 GEIA NMVOC groups. Both files include mappings between the pollution sources described by each chemical profile and the emission source categories reported by two widely used sector classification systems: the Selected Nomenclature for Air Pollutant Reporting (SNAP) and the Nomenclature for Reporting (NFR). The third file is ready-to-use dataset for air quality modelling, providing the speciation of total NMVOC emissions reported by the Copernicus Atmosphere Monitoring Service European regional emission inventory version 8.1 (CAMS-REGv8.1)^12,33^. This dataset is organised by GEIA NMVOC group, EU country, Gridded Nomenclature for Reporting (GNFR) emission sector (15 sources), and year,covering the period from 2005 to 2022.

## Methods

NMVOC speciation profiles were compiled from peer-reviewed literature, reports, established databases, and validated measurement campaigns. All data sources used to compile the NMVOC database are are documented within the database files. Examples of source-specific profiles include residential wood combustion, combining profiles for wood boilers^34^ and for fireplaces and wood stoves from the SPECIATE database^35^, solvent use^36^, coating application^37–39^, chemical products^22,40–43^, passenger cars^44^, and motorcycles^45^.

As a first step, we targeted the 153 SNAP source categories reporting anthropogenic NMVOC emissions for Spain, which allowed covering multiple combustion and transport-related activities, solvent use, agricultural sources, and industrial processes^31^. The dataset was subsequently expanded to include SNAP sources not occurring in Spain but relevant in other European countries, such as coal mining^19^. In parallel, profiles were added for NFR sectors and for fuel-specific categories. Some sectors are dominated by a single fuel or process, whereas others represent mixtures (e.g., energy production, road transport, and residential, commercial, and industrial combustion).

To address limitations in the SNAP and NFR classifications—such as sector aggregation and the lack of fuel-specific reporting—we applied a weighted-average (WA) approach (Equation (1)). This approach combines multiple sub-sector or fuel-specific profiles using weights derived from recent multi-year energy consumption statistics (2020-2024) for the EU-27^46,47^, and emissions factors from the European Monitoring and Evaluation Programme (EMEP) inventory guidebook^48^. All individual profiles used to create the WA profiles are also provided in the database, allowing users to apply custom weights if needed.

1 $${f}_{k,i}^{WA}=\sum _{j\in k}{w}_{j}\,{f}_{j,i}$$

Here, ${f}_{k,i}^{WA}$ is the weighted-average mass fraction of species *i* for activity sector *k*, *f*_*j*,*i*_ is the mass fraction of species *i* in profile *j*, and *w*_*j*_ is the normalised weight of sub-activity or process *j* within sector *k*, based on energy consumption, emissions, or other relevant statistics.

For SNAP and NFR emission source categories with multiple NMVOC profiles available in the literature, the final selection was guided by four criteria: i) representativeness of European technologies, fuels, and regulatory contexts; ii) the year of measurement, with preference given to more recent data; iii) measurement quality, considering experimental design and conditions; and iv) chemical resolution, favouring profiles with a higher level of species detail and fewer lumped or unidentified compounds. Non-European profiles were used only when no suitable Europe measurements were available, particularly for sources and fuels whose characteristics are expected to vary little across regions.

Selected profiles were then processed to ensure consistency and harmonisation prior to inclusion in the final database. This included calculating mass fractions when not directly reported, using available concentration or emission data for individual compounds. Species reported below detection limits were set to zero. When profiles referred to total VOC rather than NMVOC emissions, methane contributions were removed and the remaining species were renormalised to sum to 100 %. Each individual compound was assigned a Chemical Abstracts Service (CAS) number—a unique identifier widely used to standardise chemical nomenclature–to ensure consistent chemical identification. For grouped compounds, the CAS number of the most representative species was used to facilitate subsequent linkage to chemical mechanisms. For example, when the speciation profiles reported m,p-xylene, the CAS number of m-xylene was assigned, as it typically dominates the mixture^49^. The original profile compositions are retained to preserve source-specific chemical characteristics, although a small subset of profiles may contain intermediate- or semi-volatile organic compounds (I/SVOCs). In the current database, their contribution is generally limited: out of 130 profiles, 72 do not include any I/SVOC species, over 87 % of profiles (114) are dominated by VOCs, with more than 95 % VOC content, while only two profiles—which are linked to emission sources representing less than 2.5 % of total European NMVOC emissions—show a substantial I/SVOC fraction (35–36%). Users should take this into account when applying the profiles to NMVOC emissions.

To ensure compatibility with state-of-the-art air quality modelling frameworks^28^, all individual NMVOC species were mapped to the standard set of the 25 GEIA NMVOC groups. To ensure mass conservation, species that could not be assigned to any GEIA group were placed in the group ‘voc25: Other NMVOC’; these account for less than 0.5 % of total NMVOC emissions.

Using the final harmonised database, emissions of individual NMVOC species or GEIA groups can be derived from reported total NMVOC emissions using Equation (2):

2 $${E}_{i}=\sum _{k}{E}_{k}\times {f}_{k,i}$$

Here, *E*_*i*_ is the emission of species *i* (either individual compound or a GEIA NMVOC group), *E*_*k*_ is the total NMVOC emission from sector *k* (as reported by SNAP or NFR sector), and *f*_*k*,*i*_ is the corresponding species mass fraction provided in this work.

## Data Records

The database comprises 130 harmonised NMVOC chemical speciation profiles covering more than 900 species, compiled from 39 literature sources, including aggregated databases such as SPECIATE. The data are publicly available via the Barcelona Supercomputing Center (BSC) Dataverse^50^. The dataset is distributed in three complementary files:

1. **Species-level profile database (NMVOC_Profile_DB.xlsx):** This file provides detailed NMVOC speciation at the individual compound level and consists of three worksheets:
  **Sheet 1:** General information about the dataset and data citations.
  **Sheet 2 (Profiles DB):** The complete set of NMVOC speciation profiles, reporting mass fractions for individual species together with associated metadata. Metadata include source references and notes any data processing, simplifications, assumptions, or harmonisation steps applied.
  **Sheet 3 (Sectors Linkage):** Recommended mapping between chemical speciation profiles and SNAP and NFR sectors, including complementary profiles for further use.
2. **GEIA 25 group profile database (NMVOC_Profile_DB_GEIA25.xlsx):** This file contains the same set of profiles, metadata, and sector mappings as the species-level database, but with all NMVOC emissions aggregated into the 25 GEIA NMVOC groups. The file structure and worksheet organisation mirror those of the species-level database
3. **CAMS-REGv8.1 speciation file (CAMS-REG_v8_1_NMVOC_split.txt):** A modelling-ready dataset providing NMVOC species mass fractions aggregated by EU country, GNFR emission sector, and year (2005-2022). The data are reported for the 25 GEIA NMVOC groups and follow the standard formatting conventions of CAMS-REGv8.1^12^.

## Technical Validation

The NMVOC speciation database has been indirectly validated in previous studies through comparisons of modelled and observed concentrations of selected species, including benzene, toluene, and xylene^19,31,32^. This comparison provided an initial assessment of the database’s performance and suitability for use in chemical transport models. In^19^, the use of the updated speciation resulted in improvements in model performance, especially for benzene, for which the average normalized mean bias (NMB) across Europe was reduced from -46.1% to −27.7%. For toluene and xylene, the updated speciation significantly reduced the overestimations observed in major urban areas, with NMB values decreasing from 45–194% to approximately -60% to 0 %. For xylenes, NMB values decreased from 116–542% to -29% to 256 %. However, these results should be interpreted with caution, as the validation is indirect and influenced by multiple factors beyond the speciation profiles, including the chemical mechanism used, meteorological conditions, total NMVOC emissions, and observational uncertainties.

In addition to benzene, toluene, and xylenes, the new speciation redistributes emissions among other NMVOC species, which seem to be moving in the right direction. For example, propane and ethyne, which were previously underestimated in models^17,18,51^, show substantial increases in emissions, while higher alkanes, often overestimated, decrease. Similarly, monoterpenes and isoprenes show higher contributions from anthropogenic sources in line with previous studies^52–54^. Although these changes suggest improvements, further evaluation is needed to quantify their impact.

Building on this earlier work, we document here the associated uncertainties and quality assessment framework, and present a comparison with the currently implemented NMVOC speciation used in the CAMS-REG emission inventory^12^.

### Uncertainty and Quality Assessment of Speciation Profiles

All profiles included in the database are derived from validated measurements reported in peer-reviewed studies or established technical datasets. However, several sources of uncertainty arise when mapping individual speciation profiles to aggregated SNAP and NFR source emission categories. These uncertainties can be broadly grouped into three main categories:

1. **Regional, technological, and temporal representativeness:** Large differences in technologies, fuels, and regulations among countries or regions can limit the applicability of profiles developed outside Europe. Emission characteristics can also change over time due to technological improvements or regulatory changes (e.g., the transition from solvent-based to water-based coatings or the progressive tightening of EURO vehicle emission standards). Consequently, older profiles may not fully represent present-day European emission characteristics.
2. **Measurement and analytical limitations:** The accuracy of the profiles can be affected by instrumental or analytical limitations, including uncertainties in compound identification. High-reactivity species can be lost during sampling, storage, or analysis, leading to underestimation of compounds. Other species may fall below detection limits, co-elute during separation, or undergo partial oxidation during sampling. Field measurements may also be influences by nearby diffuse emissions, potentially biasing the inferred source profile.
3. **Attribution to emission source categories:** Mapping a single profile to an emission source category that includes multiple processes, technologies, or fuels (e.g. residential combustion from boilers or passenger car exhaust), may not fully capture the full variability within that sector. Additional differences related to operational conditions, plant size, local practices, or the presence of gas treatment and abatement technologies can further influence emission composition.

To provide users with transparent guidance on the reliability of each profile-sector mapping, these uncertainties are represented using five quality codes (Table 1), following a previously proposed methodology^11^.

**Table 1:** Quality codes used to describe the representativeness of the speciation profile mapped to SNAP or NFR emission source categories.

Quality code	Description
1	**Well matched:** European profile fully representative of the target sector, fuel, and technology
2	**Good match:** Non-European profile representative of the same fuel and broadly comparable operating conditions.
3	**Acceptable match:** Non-European profile that may differ somewhat from European conditions but remains suitable for application.
4	**Partial match:** Non-European profile that shares some characteristics with the target sector or fuel.
5	**Approximation:** Best available profile used in the absence of a closer match.

All literature sources of individual speciation profiles used to compile the dataset are cited in this manuscript^22,34–45,48,55–77^ and provided in the dataset^50^ (see the “Reference” field). The associated quality codes (as defined in Table 1), assigned at the SNAP and NFR sector level, are reported in the quality code field of the SNAP_NFR_Link sheet in the dataset. These codes describe how well a given profile represents the mapped emission sector, rather than the intrinsic quality of the measurements or the level of aggregation within that sector. This distinction is particularly important for NFR sectors that combine multiple underlying activities. For example, coating applications (2D3d) is represented as a single category in NFR, but corresponds to nine separate activities in SNAP. As a result, aggregated NFR sectors may be assigned combined quality codes ‘1;2’, indicating that some constituent activities are represented by European profiles while others rely on non-European data. Aggregated sectors assigned combined quality codes ‘1;2’, represent 39.5 % of total NMVOC emissions. The remaining emissions fall under the following quality codes: 16.1 % under code 1, 39.6 % under code 2, 3.7 % under code 3, 1.1 % under code 4, and a residual fraction under code 5. A substantial share of quality code 2 profiles originates from the SPECIATE database. Sectors relying exclusively on SPECIATE represent 28.2 % of total NMVOC emissions, of which 23.3 % is linked to manure management. For this sector, the selected profile is consistent with the recommendation in the EMEP guidelines^48^. Although alternative studies could have been used^78,79^, potentially increasing the share of quality code 1 profiles, the chosen profile was retained because it reports the same dominant species (e.g., ethanol) while covering a larger number of NMVOC species.

### Comparisons with CAMS-REGv8.1 default speciation

**Fig. 1:**
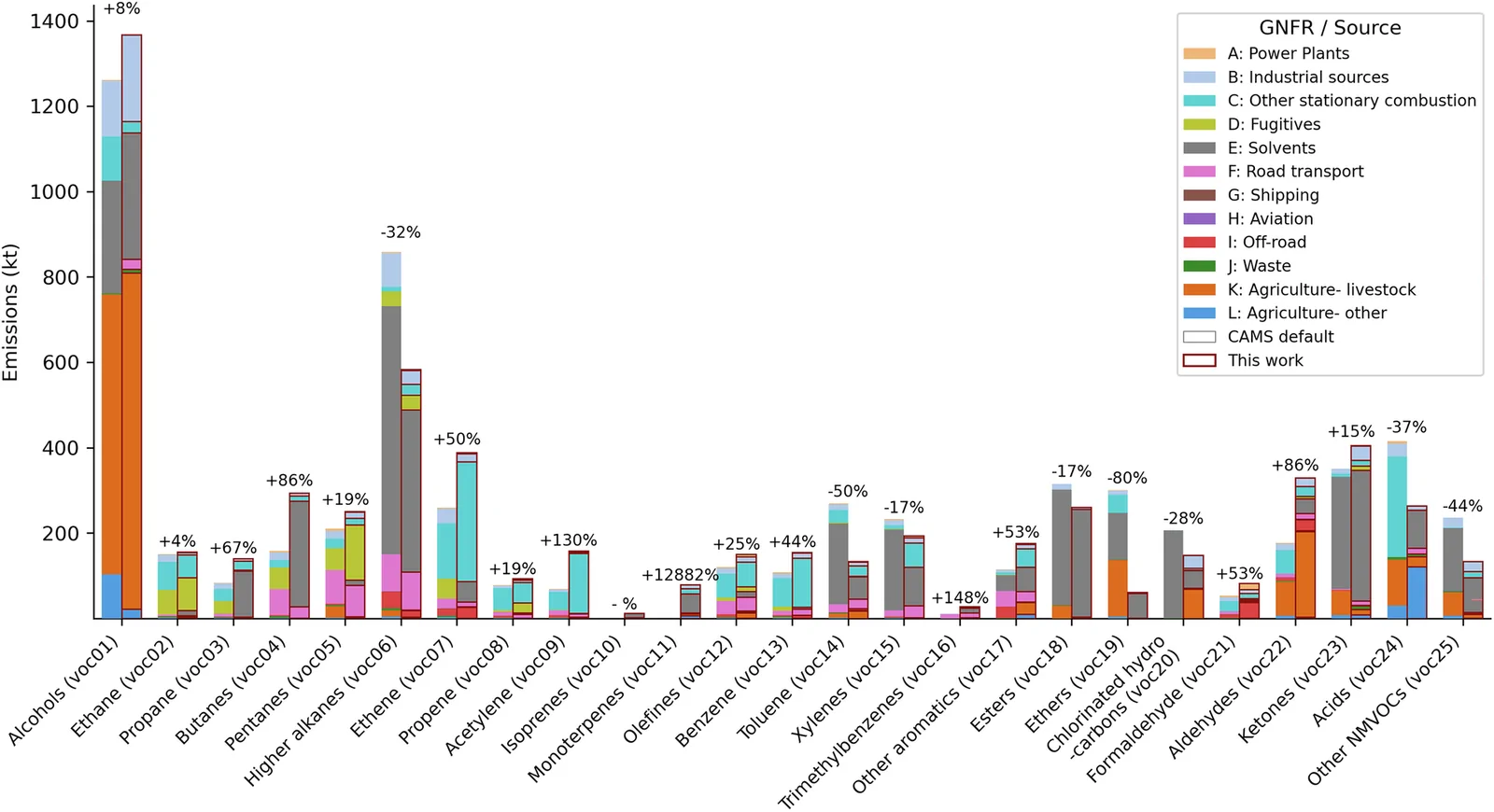
Comparison of total European NMVOC emissions in 2022 by GNFR sector for each GEIA NMVOC group (voc01-voc25), using the default CAMS-REGv8.1 speciation and the speciation developed in this work. Values above indicate the relative difference between the two speciations (CAMS default minus this work).

A comparison was carried out between the NMVOC speciation developed in this work and the default CAMS-REG speciation to evaluate differences in the distribution of GEIA NMVOC groups across GNFR sectors. The analysis uses total EU-27 emissions for 2022 from CAMS-REGv8.1 (see Fig. 1). The relative differences in total emissions for each GEIA group is presented, revealing substantial discrepancies across several species and sectors.

In both speciation datasets, alcohols (voc01) and higher alkanes (voc06) are the most abundant species. However, the third most important one differs between the two approaches, shifting from acids (voc24) in the CAMS default speciation to ketones (voc23) in the speciation developed in this work. For many species, the dominant GNFR sector remains unchanged. For example, alcohols (voc01) remain mainly associated with livestock (GNFR K) in both datasets.

In contrast, the dominant sector changes for some species. Butanes (voc04), for example, are mainly associated with fugitives (GNFR D, 32.1 %) and road transport (GNFR F, 38.6 %) in the CAMS default speciation, whereas they are largely dominated by solvents (GNFR E, 84 %) in the speciation developed in this work. The largest relative differences in total emissions occur for species traditionally associated with biogenic sources, such as isoprene (voc09) and monoterpenes (voc10). In the CAMS default speciation, these species are treated as predominantly biogenic, with only minor contributions from livestock for monoterpenes. By contrast, the speciation developed in this work redistributes these emissions across several sectors, with solvents (GNFR E) representing more than 60 % of total emissions.

**Fig. 2:**
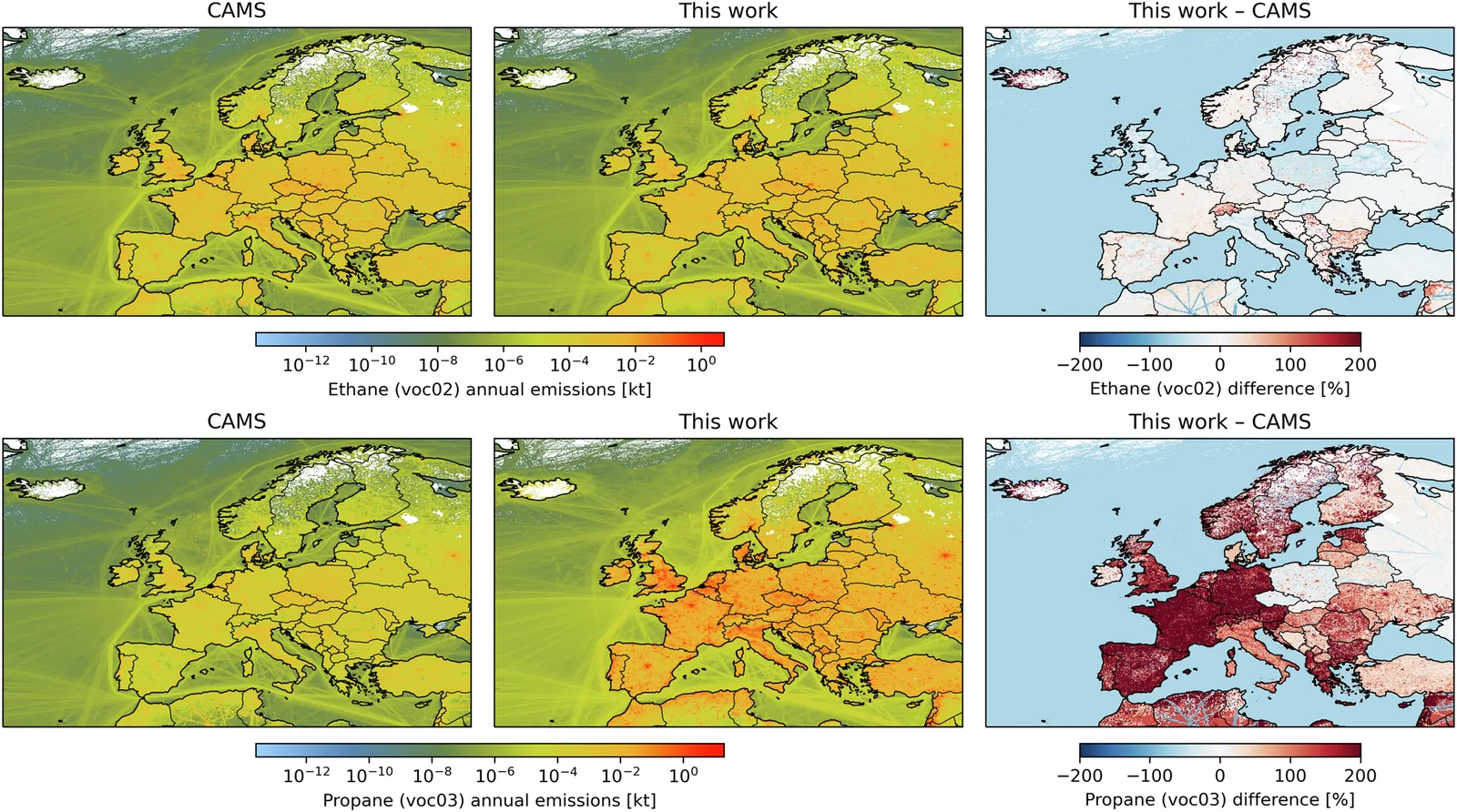
Spatial distribution of emissions in kilotonnes (kt) for selected NMVOC species in 2022 at a resolution of (0. 1^°^ × 0. 1^°^). Maps show ethane (voc02, top) and propane (voc03, bottom) based on CAMS-REGv8.1 default speciation (left) and the speciation developed in this work (centre). The right panel shows the relative differences between the two speciations.

To illustrate how updated speciation profiles impact the spatial distribution of emissions, Fig. 2 presents examples for selected explicit species included in the air quality directive, namely ethane (voc02) and propane (voc03). The maps compare the 2022 CAMS-REGv8.1 emissions using the default speciation and the updated one developed here. The resulting changes are spatially heterogeneous, with some regions showing increases while others show decreases, and in certain cases urban areas experience more pronounced changes than surrounding regions.

**Fig. 3:**
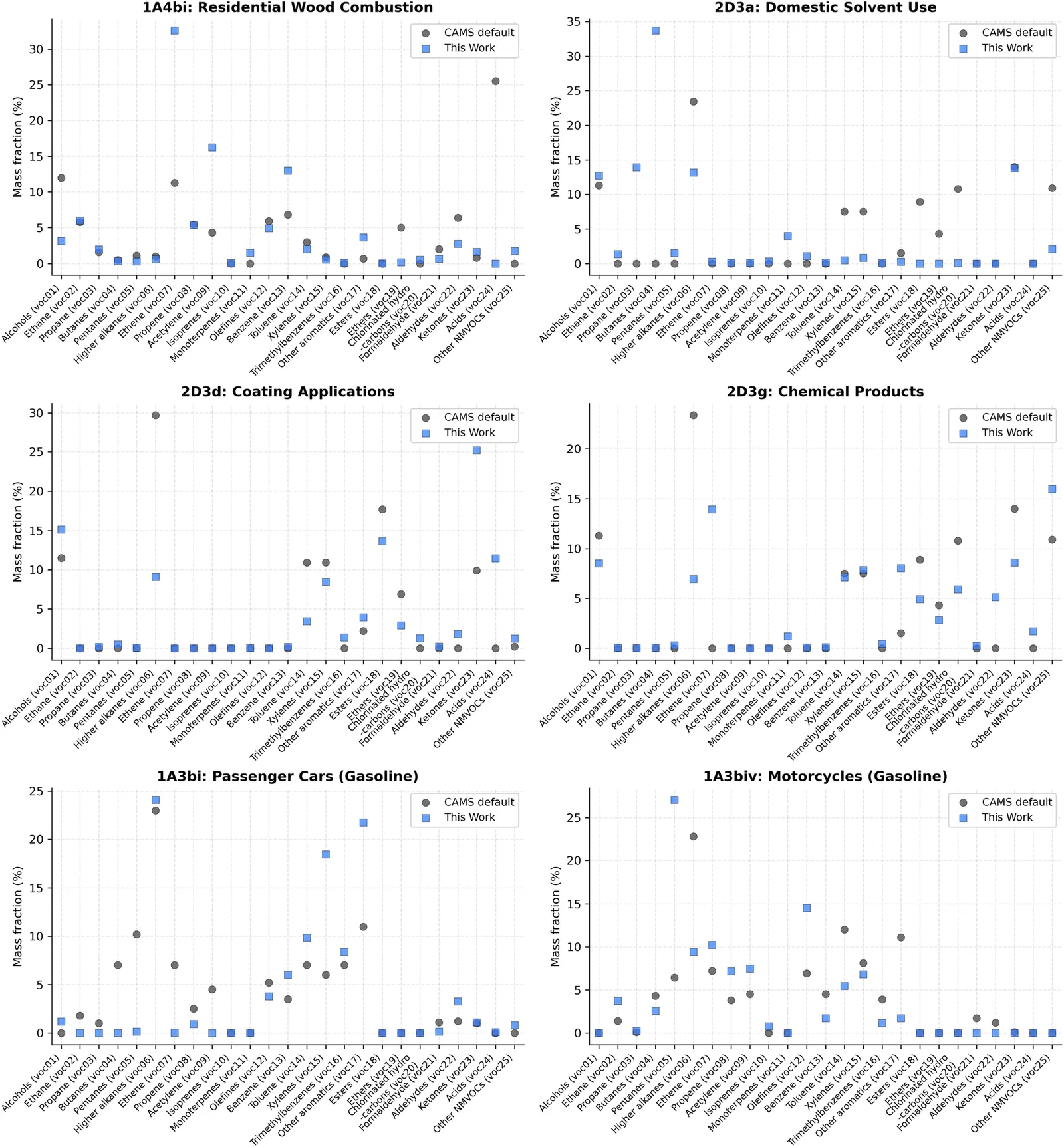
Comparison of NMVOC speciation profiles for key emission sectors, including residential wood burning (NFR 1A4bi), domestic solvents (NFR 2D3a), coating applications (NFR 2D3d), chemical products (NFR 2D3g), gasoline passenger cars (NFR 1A3bi) and motorcycles (NFR 1A3biv), between the CAMS default dataset and the profiles developed in this study.

Figure 3 compares the NMVOC speciation profiles from the CAMS default dataset and this work for selected NFR emission source categories. Together, these sectors contribute 47.6 % of total NMVOC emissions in the EU-27 in 2022. The largest contributors among them are residential combustion (NFR 1A4bi), dominated by wood burning and accounting for 22.1 % of emissions, and coating applications (NFR 2D3d) contributing 14.9 %. Marked differences are visible when different source profiles are used for the same activity, as discussed in our previous work^31^.

For passenger cars, the CAMS default speciation relies on an older profile derived from measurements of vehicles manufactured before 1999 in Germany^80^. In contrast, the speciation provided in this work is based on a more recent study that measured emissions from modern (EURO 5) passenger cars in France^44^. The exact impact of technological improvements is difficult to quantify because many factors such as fuel composition, injection technology, engine capacity, aftertreatment systems, cold temperature, driving conditions, and mileage can impact NMVOC emissions both quantitatively and qualitatively^44^.

For domestic solvent use, the CAMS default speciation is based on measurements conducted in Germany in 1995^10^, whereas this work relies on a more recent study with NMVOC measurements from 60 UK households, covering a broad set of domestic solvent-use activities^36^. The newer profiles show a higher share of NMVOCs used as propellants in aerosol products, such as propane and butane, likely because older profiles considered only non-aerosol products, while the new profiles reflect a mix of aerosol and non-aerosol use consistent with current patterns. A lower share of aromatics, such as toluene and xylene, is also observed, similar to trends in the coating application sector, reflecting the shift from solvent-based to water-based paints over time.

## Data Availability

All data was processed using Python version 3.11 and is stored in spreadsheet formats Excel .xlsx) and in plain-text files (.txt). The script, input profiles, and auxiliary files used to generate the database files are available in the repository: https://gitlab.earth.bsc.es/es/nmvoc-speciation-database-data-and-scripts.
